# Cilostazol Improves Proangiogenesis Functions in Human Early Endothelial Progenitor Cells through the Stromal Cell-Derived Factor System and Hybrid Therapy Provides a Synergistic Effect* In Vivo*

**DOI:** 10.1155/2016/3639868

**Published:** 2016-08-09

**Authors:** Shih-Ya Tseng, Ting-Hsing Chao, Yi-Heng Li, Chung-Lung Cho

**Affiliations:** ^1^Department of Biological Science, National Sun Yat-Sen University, Kaohsiung 704, Taiwan; ^2^Department of Internal Medicine, National Cheng Kung University College of Medicine and Hospital, Tainan 804, Taiwan

## Abstract

This study investigated the effect of cilostazol on proangiogenesis functions in human early endothelial progenitor cells (EPCs)* in vitro* and the therapeutic implication of hybrid therapy with cilostazol and human early EPCs* in vivo*. Cilostazol significantly increased colony-forming units and enhanced differentiation of EPCs toward endothelial lineage. Treatments resulted in antiapoptotic effects and stimulated proliferation and migration and* in vitro* vascular tube formation through activation of stromal cell-derived factor-1 (SDF-1)/C-X-C chemokine receptor type 4 (CXCR4)/phosphatidylinositol-3 kinase (PI3K)/Akt signaling pathway. Blood flow recovery and capillary density in murine ischemic hindlimbs were significantly improved in cilostazol-treated, human early EPCs-treated, and cotreatment groups. The effects were attenuated with SDF-1*α* inhibition. Plasma SDF-1*α* levels were significantly higher in 3 active treatment groups after surgery, with greatest effects observed in hybrid therapy. The angiogenic effects of transplanted EPCs pretreated with cilostazol* ex vivo* were superior to untreated EPCs using* in vivo* Matrigel assay. Implanted EPCs were incorporated into the capillary, with pretreatment or cotreatment with cilostazol resulting in enhanced effects. Taken together, cilostazol promotes a large number of proangiogenic functions in human early EPCs through activation of SDF-1/CXCR4/PI3K/Akt signaling, and hybrid therapy provides a synergistic effect* in vivo*. Cotreatment may be beneficial in ischemic disease.

## 1. Introduction

Critical limb ischemia (CLI) is an advanced form of peripheral artery disease in which the narrowing arteries limit blood supply to other organs of lower extremities with resultant resting pain and tissue loss [1]. For nearly a decade, proangiogenic stem cell therapy has been anticipated as a promising therapeutic strategy in patients with ischemic cardiovascular disease that have no other surgical or endovascular options for revascularization [1–3]. However, their therapeutic application has been limited by their inability to yield a sufficient number of stem cells, as well as impaired regeneration capacity such as mobilization, homing, and graft survival due to aging, preexisting multiple cardiovascular risk factors, and harsh ischemic environments [1–3].

Blood vessel growth is mediated by angiogenesis, which is defined as the formation of new blood vessel out of existing vessels. The process of angiogenesis includes migration and proliferation of endothelial cells (ECs), extracellular matrix degradation, and capillary tube formation, and vasculogenesis is a* de novo* process where circulating progenitor cells contribute to adult neovascularization [2, 4, 5]. Some angiogenic factors, for example, stromal cell-derived factor-1*α* (SDF-1*α*), have been implicated in the neovascularization on the vascular endothelium for bone marrow-derived circulating endothelial progenitor cells (EPCs) [6, 7]. SDF-1*α* is a major angiogenic factor that plays an important role in the recruitment and retention of C-X-C chemokine receptor type 4- (CXCR4-) positive bone marrow cells, such as EPCs [7], to the neo-angiogenic niches supporting neovascularization for improving perfusion of ischemic tissue [8, 9].

Some studies have revealed that administration of expanded EPCs, with or without CXCR4 gene transfer, to animal models of hindlimb ischemia and acute myocardial infarction could improve blood flow and subsequent functional recovery, documented as limb salvage and improvement of myocardial function mediated through SDF-1*α*/CXCR4 pathway [7, 10]. However, the circulating number of EPCs and endogenous angiogenic factors in CLI patients are usually lower than healthy volunteers [11]. Owing to the potential therapeutic limitations described previously, the investigation of an efficient cell therapy strategy is crucial to obtaining a better therapeutic outcome in patients with peripheral artery disease.

Cilostazol, a selective phosphodiesterase 3 inhibitor, is a commercially available drug that provides antiplatelet and vasodilatory effects mediated by an increase in intracellular cyclic adenosine monophosphate levels [4, 12, 13] and is prescribed to patients with peripheral artery disease only with intermittent claudication. However, there is still a lack of evidence supporting the benefits of this compound in CLI. Notably, cilostazol could provide additional cellular and proangiogenic effects, including protection of ECs from apoptosis [4, 12, 14], stimulation of the release of angiogenic factors [4, 15], and improved endothelial function of ECs [4, 12], thereby potentially enhancing angiogenesis [4, 12, 16]. However, the effects of cilostazol on the functions of human early EPCs and its potential mechanisms related to SDF-1*α*/CXCR4 pathway are not well understood. Lastly, the role of hybrid therapy with transplantation of human early EPCs and cilostazol in hindlimb ischemia has never been reported.

In the present study, we have investigated the effects of cilostazol on multiple proangiogenic functions in human early EPCs, including proliferation, migration,* in vitro* vascular tube formation, antiapoptosis, and differentiation potential toward endothelial lineage, as well as secretion and expression of SDF-1*α*. We also studied the effects of hybrid therapy, with transplantation of human early EPCs and cilostazol, on hindlimb ischemia and* in vivo* Matrigel angiogenesis. The mechanisms involving the SDF-1/CXCR4/phosphatidylinositol-3 kinase (PI3K)/Akt signaling pathway were also examined.

## 2. Materials and Methods

All volunteers provided signed informed consent and this study followed the regulation of the Institutional Review Board of the National Cheng Kung University Hospital. All the* in vitro* experiments were performed in the EPCs from healthy donors without any traditional coronary risk factors.

### 2.1. Reagents

Human vascular endothelial growth factor (VEGF), human basic fibroblast growth factor (bFGF), human epidermal growth factor (EGF), insulin growth factor (IGF), M199 medium, fetal bovine serum (FBS), 4′,6-diamidino-2-phenylindole (DAPI), cell dissociation buffer, and phosphate buffered saline (PBS) were purchased from Invitrogen (Grand Island, NY, USA). Cilostazol, lectin, SDF-1*α*, LY294002 (a PI3K inhibitor), Akt inhibitor (Akti), AMD3100 (a CXCR4 inhibitor), Giemsa, crystal violet reagent, dimethyl sulfoxide (DMSO), fibronectin, Drabkin reagent 525, and Triton X-100 were purchased from Sigma-Aldrich (St. Louis, MO, USA). Ficoll-Paque Plus was purchased from Amersham Pharmacia Biotech AB (Uppsala, Sweden). The mouse-anti-human antibodies specific to CD45-conjugated with peridinin chlorophyll protein (Per-CP), CD34 with fluorescein isothiocyanate (FITC), and CD146 with phycoerythrin (PE) were purchased from BD Pharmingen (Franklin Lakes, NJ, USA) and KDR-conjugated (VEGF-receptor 2; VEGF-R2) with PE was from R&D Systems (Minneapolis, MN, USA). Antibodies against phospho-Akt (Ser437) and Akt were purchased from Cell Signaling Technology (Danvers, MA, USA). Antibodies against phosphoendothelial nitric oxide synthase (eNOS) (Ser1177) and eNOS were from BD Transduction Laboratories (Franklin Lakes, NJ, USA), whereas antibodies against CXCR4 and SDF-1*α* were purchased from Santa Cruz Biotechnology (Santa Cruz, CA, USA). Rabbit-against-human anti-actin antibody, mouse anti-human SDF-1*α* neutralizing monoclonal antibody, IgG control, and polyvinyldifluoride (PVDF) membranes were purchase from Millipore (Billerica, MA, USA). A 5-bromo-2′-deoxyuridine (BrdU) kit and a cell death detection enzyme-linked immunosorbent assay (ELISA) kit were purchased from Roche Diagnostic GmbH (Mannheim, Germany). Matrigel and a rat monoclonal antibody against murine CD31, CD34, and CD45 were purchased from BD Biosciences (San Jose, CA, USA). Antibody against human VEGF-R2 and CD31 as well as biotinylated rabbit anti-rat secondary antibody, 3-amino-9-ethylcarbazole (AEC), and streptavidin-horseradish peroxidase (HRP) were purchased from DAKO (Glostrup, Denmark). DiI-acetylated low density lipoprotein (DiI-acLDL) was purchased from Biomedical Technologies (MA, USA). Sodium dodecyl sulfate polyacrylamide gels for electrophoresis (SDS-PAGE) were purchased from Bio-Rad Laboratories (Hercules, CA, USA).

### 2.2. Culture and Characterization of EPCs

Human peripheral blood mononuclear cells (PBMCs) were isolated and cultured as previously described [4, 8, 12, 17]. Briefly, mononuclear cells were isolated by Ficoll-Paque density gradient centrifugation and cultured on fibronectin-coated culture plates. After centrifugation, isolated cells were maintained in M199 medium supplemented with 20% (v/v) FBS, 10 ng/mL VEGF, 2 ng/mL bFGF, 10 ng/mL EGF, and 2 ng/mL IGF. Cilostazol or related inhibitors were added for the colony formation assay or immunofluorescence assay. After 3 days in culture, nonadherent cells were removed and new medium was applied. After 6 days in culture, early EPCs were confirmed by uptake of DiI-acLDL and lectin. Cilostazol or related inhibitors were then added to the wells in assays for proliferation, migration, antiapoptotic effects, and* in vitro* vascular tube formation by early EPCs. The selected dose of cilostazol was used according to our previous studies [4, 12].

The characteristics of human early EPCs were identified by flow cytometry analysis as described previously [12]. In brief, 2 × 10^5^ cells were incubated with antibodies specific to CD45-conjugated with Per-CP, CD34 with FITC, KDR with PE, and CD146 with PE. Fluorochrome-conjugated isotype identical antibodies served as controls. After incubation for 15 minutes, cells were washed and subsequently fixed. In total, 10,000 events were collected on a FACSCanto flow cytometer (BD Pharmingen, Franklin Lakes, NJ, USA) and analyzed by gating appropriate cell populations plotted on forward scatter and side scatter. The percentages of cells positive for KDR, CD34, and CD146 were further identified while the CD45^dim^ subpopulation was gated.

### 2.3. Identification of Differentiation of EPCs toward Endothelial Lineage

To further identify the stimulatory effect of cilostazol on the differentiation of EPCs toward endothelial lineage, immunofluorescence staining was performed to stain VEGF-R2 and CD31 endothelial surface markers, as described previously [4, 12]. In brief, after culture for 6 days, cells were fixed with 4% paraformaldehyde for 15 minutes. After permeabilization with 0.1% Triton X-100 in PBS for 10 minutes, cells were rinsed with PBS three times and then incubated with FITC-labeled antibody against VEGF-R2 and PE-conjugated antibody against CD31 for 2 hours. The stained cells were quantified with an Olympus CKX31 inverted microscope (Tokyo, Japan) at a magnification of 100x. Three random fields of cells were counted using MetaMorph imaging software (Molecular Devices Inc., Downingtown, PA, USA).

### 2.4. Colony Formation by Human Early EPCs

Colony formation by human early EPCs was evaluated as previously described [4]. In brief, PBMCs (1 × 10^6^ cells) were cultured on fibronectin-coated 24-well plates and incubated in medium with various concentrations of cilostazol, SDF-1*α* (100 ng/mL), LY294002 (10 *μ*M), Akti (10 *μ*M), or AMD3100 (2 *μ*g/mL). After 48 hours, the nonadherent cells were collected and 1 × 10^6^ cells were replated onto fibronectin-coated 24-well plates. Culture medium was changed every 3 days. After 7 days, the cells were fixed with 4% paraformaldehyde and stained with crystal violet reagent for 50 minutes. The colony densities were quantified with the Olympus CKX31 microscope at 100x magnification using MetaMorph imaging software. All of the* in vitro* experiments were performed in quadruplicate and repeated 3 times.

### 2.5. Proliferation Assay in Human Early EPCs

Cell proliferation was evaluated by measuring BrdU incorporation into cells using the Cell Proliferation ELISA Kit according to the manufacturer's protocol, as described previously [4, 12]. In brief, human early EPCs were plated at a density of 5 × 10^3^ cells/100 *μ*L in a 96-well fibronectin-coated plate incubated with cilostazol, inhibitors, mouse anti-human SDF-1*α* monoclonal antibody (10 *μ*g/mL), or IgG control in M199 medium containing 1% FBS for 24 hours. Then, BrdU (10 *μ*M) was added to the medium and the incubation was continued for an additional 4 hours. After incubation, the cells were fixed and the light absorbance of the samples was determined using a spectrophotometer (Thomas Scientific, CA, USA) with a wavelength set at 450 nm.

### 2.6. Migration Assay in Human Early EPCs

The chemotactic motility of EPCs was measured using Transwell with filters (8 *μ*m pore size; Costar, USA) as previously described [4, 12]. The lower filter membranes were coated with fibronectin overnight at 4°C. Various concentrations of cilostazol, inhibitors, mouse anti-human SDF-1*α* monoclonal antibody, or IgG control in M199 medium were placed in the lower wells. SDF-1*α* served as a positive control. EPCs (1 × 10^5^ cells in 100 *μ*L) were suspended in M199 medium supplemented with 0.5% serum and placed onto each upper well. The Transwell system was then incubated at 37°C for 24 hours. Cells were then fixed with 4% paraformaldehyde and stained with Giemsa solution. Cells migrating into the lower chamber were counted in 5 random microscopic fields per membrane using the Olympus CKX31 microscope with the Image-Pro Plus software (Media Cybernetics, Bethesda, MD, USA).

### 2.7. Apoptosis Assay in Human Early EPCs

Human early EPCs were plated at a density of 1 × 10^4^ cells/100 *μ*L in a 96-well plate and incubated with various concentrations of cilostazol and inhibitors in M199 medium containing 1% FBS for induction of apoptosis for 24 hours. A quantitative ELISA for detection of nucleosome fragments was used in accordance with the manufacturer's instructions.

### 2.8. *In Vitro* Vascular Tube Formation Assay in Human Early EPCs

A Matrigel tube formation assay was performed to assess the ability of EPCs to form vascular structures. A total of 200 *μ*L Matrigel was added onto each well of a 48-well plate and polymerized for 30 minutes at 37°C. EPCs containing various concentrations of cilostazol and inhibitors were placed in the Matrigel-coated wells at a density of 2.5 × 10^4^ cells per well in M199 medium containing 20% FBS. The plates were incubated for 5 days at 37°C in a 5% CO_2_ humidified atmosphere. Images were captured with an IX71 inverted microscope (Olympus, Tokyo, Japan) and the total length of the tubules per well in five randomly selected fields (100x) was measured by ImageJ software (National Institutes of Health, Bethesda, MD, USA).

### 2.9. Determination of SDF-1*α* and CXCR4 in EPCs by Western Blot Analysis

To detect cilostazol-stimulated expression of CXCR4 and SDF-1*α* proteins, EPCs were preincubated with various concentrations of cilostazol in M199 supplemented with 0.5% serum for 24 hours before protein harvesting. Proteins were separated by SDS-PAGE (10% polyacrylamide gel) and transferred to PVDF membranes, which were preincubated with antibodies against CXCR4, SDF-1*α*, and actin. Antibody binding was detected with HRP-conjugated anti-rabbit IgG (Jackson ImmunoResearch Laboratories Inc., West Grove, PA, USA) and enhanced chemiluminescence (PerkinElmer, Shelton, CT, USA), followed by exposure to X-ray film.

### 2.10. Measurement of SDF-1*α* Secreted from Human Early EPCs in Culture Medium

To determine cilostazol-stimulated secretion of SDF-1*α* proteins, EPCs were preincubated with various concentrations of cilostazol in M199 medium supplemented with 0.5% serum for 24 hours. The culture medium was aspirated and kept at −80°C until assay. SDF-1*α* levels in the culture medium were measured using an SDF-1*α* ELISA kit according to the manufacturer's instructions (R&D Systems, Minneapolis, MN, USA).

### 2.11. Preparation of Administered Reagents, Transplanted Cells, and Transplantation Procedure

Cilostazol was dissolved in DMSO and diluted with saline to a 20% solution immediately before use. The selected dose of cilostazol and number of transplanted cells were used according to our previous studies [4, 12] and other reports [17]. For preparation and transplantation of human early EPCs, PBMCs and subsequent adherent cells were cultured in M199 medium for 6 days and were detached by cell dissociation buffer for 30 minutes. EPCs (2 × 10^5^) were washed and resuspended in 50 *μ*L of PBS.

Mice were randomized into four groups (*n* = 6 in each group): (1) cilostazol group: it combined both administration of cilostazol (10 mg/kg) by intraperitoneal (IP) injection 30 minutes prior to surgical induction of ischemia and twice per day thereafter at the same dose from days 2 to 7 and intramuscular (IM) injection of 50 *μ*L PBS over 3 different sites of the calf muscle in ischemic hind limbs using a 29 G needle immediately after operation; (2) EPCs group: cell pallet (2 × 10^5^ EPCs in 50 *μ*L) was separately injected into 3 different sites of the calf muscle in the ischemic hind limbs immediately after operation and IP injection of DMSO (equivalent volume as cilostazol group) 30 minutes prior to inducing ischemia and twice per day thereafter at the same dose from days 2 to 7; (3) hybrid group: it combined both IM injection of 2 × 10^5^ culture-expanded human early EPCs and IP injection of cilostazol (10 mg/kg) following the same schedule listed in groups (1) and (2) above; (4) vehicle group: it combined injection of DMSO IP and cell-free PBS IM into peritoneum and ischemic hindlimb muscle at the aforementioned time points, respectively.

### 2.12. Hindlimb Ischemia in Experimental Animals

The procedure to induce hindlimb ischemia has been described in detail previously [4, 12]. Briefly, 5-week-old severe combined immunodeficient (SCID) mice were anesthetized with an IP injection of pentobarbital (80 mg/kg). The right femoral vein was exposed and ligated. A segment from the proximal end of the right femoral artery to the saphenopopliteal bifurcation and the right popliteal artery were then ligated and excised. The skin was closed by using a surgical stapler.

All procedures in mice were conducted in accordance with the Principles of Laboratory Animal Care published by the National Institute of Health and the National Cheng Kung University Standards for Animal Care.

### 2.13. Laser Doppler Perfusion Imaging (LDPI)

The measurement of hindlimb blood flow was performed with a LDPI analyzer (Moor Instruments, Devon, UK), as previously described [4, 12]. After anesthesia, mice were placed on a heating plate at 40°C for monitoring perfusion by serial scanning of surface blood flow of the hind limbs on days 0 (before and immediately after operation), 3, 7, 10, 14, 21, and 28 after surgery. The perfusion ratio (%) was calculated as the ratio of blood flow on the ipsilateral compared to the contralateral side.

### 2.14. Measurement of Plasma Level of SDF-1*α* in Mice

Blood samples were collected through cardiac puncture 24, 48, and 72 hours after inducing hindlimb ischemia, and plasma samples were kept at −80°C until assay. The plasma level of SDF-1*α* was determined using an SDF-1*α* ELISA kit according to the manufacturer's instructions (R&D Systems, Minneapolis, MN, USA).

### 2.15. Quantification of CD34^+^CD45^dim^ Cells by Flow Cytometry

We counted CD34^+^CD45^dim^ cells in peripheral blood according to the International Society of Hematotherapy and Graft Engineering (ISHAGE) guidelines [4, 12, 18]. Briefly, 50 *μ*L of whole blood obtained from mouse tail veins was incubated with PE-conjugated anti-CD34 and FITC-conjugated anti-CD45 monoclonal antibodies in a TruCOUNT tube (BD Pharmingen, San Jose, USA) for 30 minutes. CD34^+^CD45^dim^ cells were then analyzed and quantified on a FACSCanto flow cytometer according to the ISHAGE sequential gating strategy.

### 2.16. Measurement of Capillary Density in Ischemic Limbs

Measurement of capillary density in ischemic limbs has been described in detail previously [4, 12]. The mice were sacrificed 28 days following surgery, just after the final LDPI procedure and blood collection. Whole limbs from experimental mice were fixed in 4% paraformaldehyde at 4°C overnight, then embedded in an optimal cutting temperature compound (Sakura, Tokyo, Japan) after being immersed in a 30% sucrose solution, and subsequently frozen at −80°C. Cryostat sections (5-*μ*m thick) were prepared and incubated with rat anti-mouse CD31 antibody overnight at 4°C. Sections were washed and incubated with biotinylated rabbit anti-rat secondary antibody at a 1 : 500 dilution for 30 minutes. Streptavidin-HRP was added for 10 minutes, and color development was performed by addition of the AEC substrate. Capillary density was quantified using an Olympus IX71 inverted microscope by counting the mean number of capillaries, as revealed by positive expression of CD31 on ECs.

### 2.17. Western Blot Analysis of Ischemic Muscle Tissue

Homogenates of muscle tissues were prepared and proteins (40 *μ*g per lane) were separated by SDS-PAGE (10% polyacrylamide gel) and transferred to PVDF membranes. Membranes were incubated with antibodies against phospho-eNOS, eNOS, phospho-Akt, Akt, SDF-1*α*, and CXCR4. Antibody binding was detected with HRP-conjugated secondary antibodies (Jackson ImmunoResearch Laboratories Inc., West Grove, PA, USA) and enhanced chemiluminescence (PerkinElmer, Shelton, CT, USA), followed by exposure to X-ray film.

### 2.18. *In Vivo* Inhibition of SDF-1*α* in Mice

Anti-SDF-1*α* neutralizing antibody or control IgG was injected intraperitoneally. Mice were randomized into four groups (*n* = 3 in each group): cilostazol treatment alone, EPC transplantation alone, hybrid therapy, and vehicle control. After inducing hindlimb ischemia, mice were treated with IP injections of 32 *μ*g of anti-SDF-1*α* neutralizing antibody diluted in PBS twice weekly for 4 weeks after surgery as previously described [19]. Injections of corresponding volume of control IgG served as a background control.

### 2.19. *In Vivo* Matrigel Angiogenesis Model

An* in vivo* Matrigel angiogenesis model was used to assess angiogenic effects as previously described [20]. Early EPCs were labeled with DiI-acLDL before mixing with Matrigel. In brief, growth factor-reduced liquid Matrigel (0.5 mL) containing EPCs only (1 × 10^6^ cells), EPCs (1 × 10^6^ cells) pretreated with cilostazol (30 *μ*M) for 24 h, or combined EPCs (1 × 10^6^ cells) and cilostazol (30 *μ*M) was subcutaneously injected into a 5-week-old SCID mouse near the abdominal midline. Matrigel with DMSO/PBS served as the negative control. Fourteen days after injection, mice were euthanized, and the Matrigel plaques were surgically removed. For macroscopic analysis of angiogenesis, hemoglobin content in Matrigel was measured with Drabkin reagent 525. For histological analysis, rat anti-mouse CD31 antibody was used. The same specimens were used for hematoxylin and eosin staining. Immunofluorescence staining was performed to examine the incorporation of DiI-acLDL-labeled EPCs with different treatment strategies into the neovascularization sites. The results were the mean of six subjects, and the experiments were performed thrice.

### 2.20. Statistical Analysis

All data are expressed as mean ± standard error of the mean (SEM) from at least 3 independent experiments. Differences between the treatment groups were evaluated using Student's* t*-test followed by Fisher's analysis or ANOVA for comparisons between two or more means. Values of *P* < 0.05 were considered significant.

## 3. Results

### 3.1. Phenotypical Characterization of EPCs

**Figure 1:**
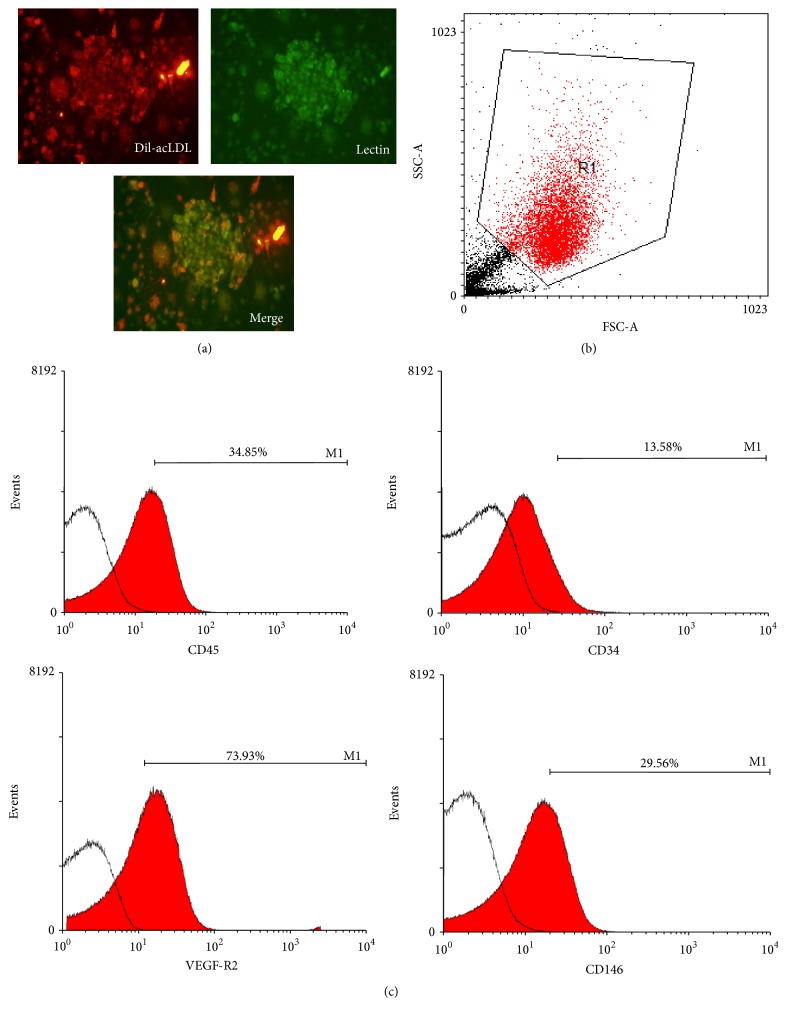
Phenotypical characterization of human early EPCs. (a) Representative figure showing double staining of DiI-acLDL (red) and FITC-labeled lectin (green) in cultured human early EPCs. Original magnification is 200x. The length of the bar is 100 *μ*m. (b) Gating the target cell population by flow cytometry analysis. (c) The percentages of cells positive (the red area in the event plots) for CD45, CD34, VEGF-R2, and CD146. The surface markers were identified while the CD45^dim^ subpopulation was gated and the isotype IgG control (the blank area in the event plots) was adjusted. DiI-acLDL, DiI-acetylated low density lipoprotein; VEGF-R2, vascular endothelial growth factor receptor type 2.

The phenotypes of human early EPCs were characterized first. The early EPCs showed round-spindle shaped morphology (Figure 1(a)). These cells could uptake acLDL and lectin to a degree compatible with the characteristics of early EPCs (Figure 1(a)). A flow cytometry analysis of a gated target population (Figure 1(b)) showed that cultured EPCs expressed a smaller percentage of CD45, a pan-leukocyte surface marker, and were strongly positive for KDR, to a lesser extent CD34 and CD146, while the CD45^dim^ subpopulation was gated (Figure 1(c)). Transplantation of EPCs into ischemic hind limbs could participate in formation of perfused capillaries* in vivo*, partly by direct incorporation into existing blood vessels; this angiogenic potential was later confirmed in mice. These results indicated that the isolated cells behaved like early EPCs.

### 3.2. Cilostazol Promotes Expression of Endothelial Lineage Markers in EPCs

**Figure 2:**
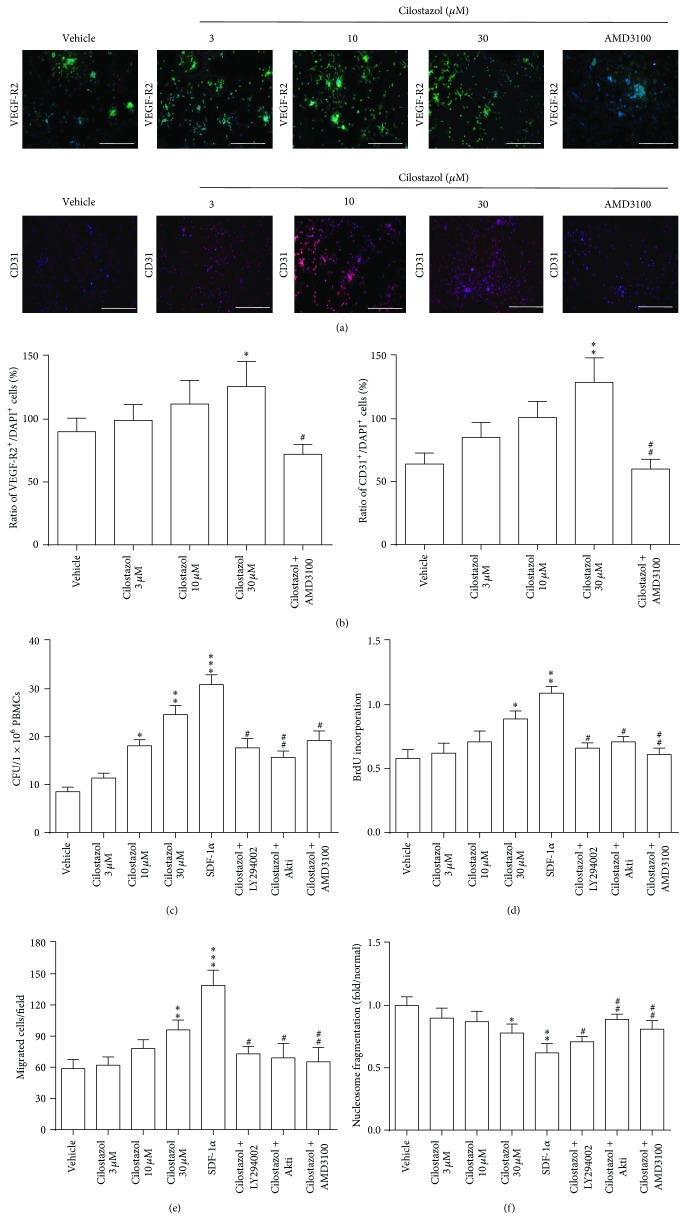
Stimulating effects of cilostazol on the expression of endothelial lineage markers and angiogenesis functions in human early EPCs. (a) Representative images of the expression of VEGF-R2 (green) and CD31 (red) by immunofluorescence staining. PBS treatment was performed as a vehicle control. A CXCR4 inhibitor, AMD3100, was used to suppress the effect of cilostazol with highest dose (30 *μ*M). The length of the bar is 100 *μ*m. (b) Quantification results for the expression of VEGF-R2 or CD31 after treatment with different doses of cilostazol. The stimulating effect of cilostazol could be attenuated with AMD3100. Results were shown in ratio of VEGF-R2^+^ (CD31^+^)/DAPI^+^. (c) SDF-1*α* was used as a positive control. Cilostazol enhanced colony formation (c), proliferation (d), migration (e), and antiapoptosis (f) in human early EPCs. These effects were reversed by inhibitors. ^*∗*^ *P* < 0.05, ^*∗∗*^ *P* < 0.01, and ^*∗∗∗*^ *P* < 0.001, significantly different compared with vehicle control. ^#^ *P* < 0.05 and ^##^ *P* < 0.01, significantly different compared with cilostazol-treated (30 *μ*M) cells alone. BrdU, 5-bromo-2′-deoxyuridine; CFU, colony-forming unit; DAPI, 4′,6-diamidino-2-phenylindole; PBMCs, peripheral blood mononuclear cells; SDF-1*α*, stromal cell-derived factor-1*α*; VEGF-R2, vascular endothelial growth factor receptor type 2.

To determine the effect of cilostazol treatment on the differentiation of EPCs toward endothelial lineage, the early EPCs were treated with vehicle, 3, 10, or 30 *μ*M of cilostazol for 24 h, and the expressions of VEGF-R2 and CD31 were subsequently analyzed by immunofluorescence staining (Figure 2(a)). Our data showed that expressions of VEGF-R2 and CD31 (normalized to DAPI-positive cells) were significantly higher in EPCs treated with 30 *μ*M of cilostazol than vehicle-treated cells (141.2 ± 13% versus 99.6 ± 9%, *P* < 0.05; 93.7 ± 12% versus 67.3 ± 7%, *P* < 0.01, resp.). Furthermore, this stimulating effect was attenuated by AMD3100 (Figure 2(b)). These results indicated that cilostazol treatment dose-dependently promoted the differentiation of human early EPCs toward endothelial lineage mediated through CXCR4 system.

### 3.3. Cilostazol Enhances Angiogenesis Functions in Early EPCs

We then tested whether cilostazol would affect the angiogenesis functions of early EPCs such as colony formation, cell proliferation, migration, and antiapoptotic effects. Cilostazol treatment dose-dependently increased the colony-forming units of EPCs with statistical significance achieved by a dose equal to or higher than 10 *μ*M (Figure 2(c)). Cilostazol could also promote cell proliferation and migration and provide antiapoptotic effects in a dose-dependent manner, with greatest effect obtained at the highest dosage (30 *μ*M) (Figures 2(d)–2(f)). The above angiogenesis functions of human early EPCs were also improved with SDF-1*α*, as expected. Previous studies have indicated that SDF-1*α* induces cell proliferation and migration in early EPCs through activation of PI3K-Akt pathway [9]. Therefore, inhibitors involving theses signaling pathways were used to test the mechanism of cilostazol in our assays. Our data showed that all inhibitors, including LY294002, Akti, and AMD3100, could significantly abolish the angiogenesis effect of cilostazol on colony formation, cell proliferation, migration, and apoptosis in human early EPCs compared to uninhibited positive controls (Figures 2(c)–2(f)). Taken together, these findings imply that cilostazol may provide beneficial effects on EPCs functions mediated through activation of CXCR4/PI3K/Akt pathway.

### 3.4. Cilostazol Enhances Expression of Homing-Related Proteins and* In Vitro* Vascular Tube Formation

**Figure 3:**
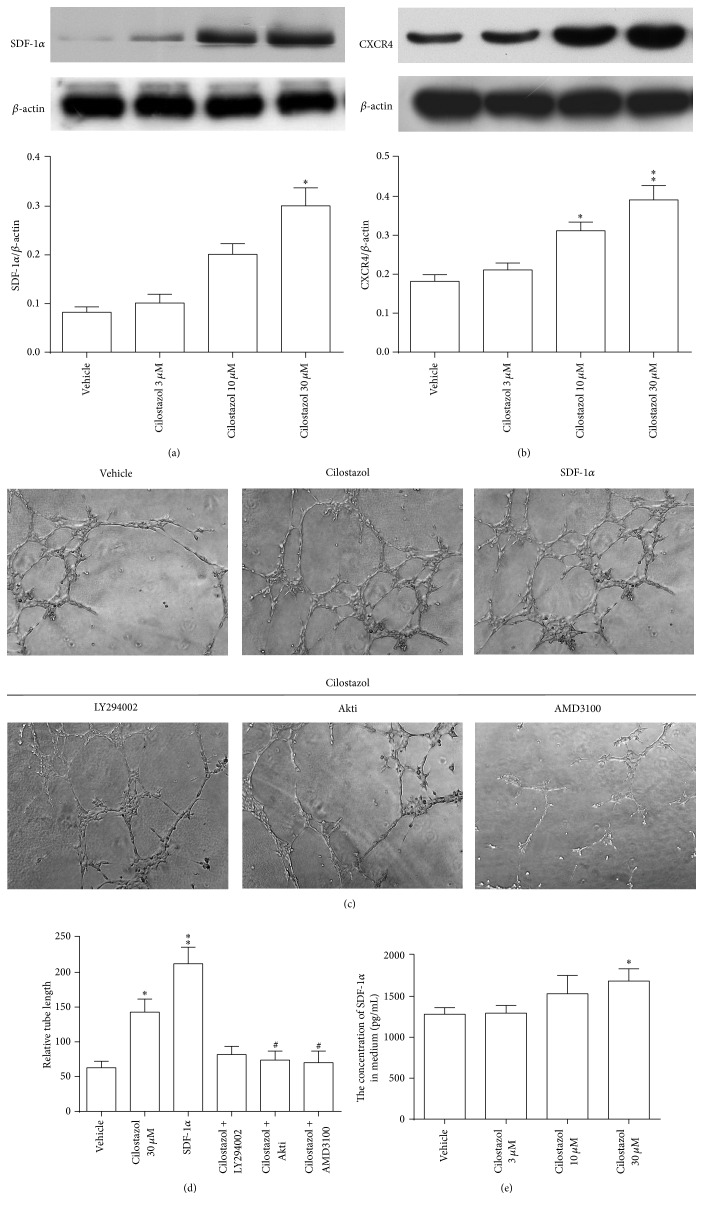
Stimulating effect of cilostazol on the expression of SDF-1*α*, CXCR4, and capillary-like vascular tube formation by human early EPCs. Representative images (upper panels) show protein expression of SDF-1*α* (a) and CXCR4 (b) detected by Western blot analysis. A quantitative analysis (lower panels) revealed that cilostazol-stimulated protein expression of SDF-1*α* (a) and CXCR4 (b) in a dose-dependent manner with peak improvement at 30 *μ*M cilostazol. (c) Illustration of early EPCs cultured on Matrigel to form a capillary-like tube network for 5 days. The effect could be enhanced with cilostazol (30 *μ*M) and SDF-1*α*. The beneficial effect of cilostazol was significantly suppressed by coincubation with LY294002, Akti, and AMD3100. Original magnification is 100x. (d) Quantification results of capillary-like structures formed in cilostazol treatment with or without inhibitors. (e) Measurement of concentration of SDF-1*α* in culture medium of EPCs treated with different dose of cilostazol. ^*∗*^ *P* < 0.05 and ^*∗∗*^ *P* < 0.01, significantly different compared with vehicle control. ^#^ *P* < 0.05, significantly different compared with cilostazol-treated (30 *μ*M) cells alone. Akti, Akt inhibitor; CXCR4, C-X-C chemokine receptor type 4; SDF-1*α*, stromal cell-derived factor-1*α*.

The expression of SDF-1*α*, a crucial growth factor for EPC differentiation, proliferation, migration, and homing, and its binding receptor, CXCR4, in human early EPCs was determined by Western blot analysis. EPCs treated with escalating doses of cilostazol displayed upregulation of SDF-1*α* and CXCR4 in a dose-dependent manner (Figures 3(a) and 3(b)). Quantitative analysis showed a significantly higher expression of CXCR4 protein at a concentration of 10 *μ*M or 30 *μ*M cilostazol than vehicle control (Figures 3(a) and 3(b)). Since 30 *μ*M cilostazol provided the most significant effect, this dose was used in the following* in vitro* experiments.

Human early EPCs cultured on Matrigel formed a capillary-like vascular tube network (left upper panel in Figure 3(c)), which became denser with cilostazol and SDF-1*α*. However, the stimulatory effects of cilostazol on capillary-like vascular tube networks formed by EPCs could be attenuated with Akti and AMD3100, and to a lesser extent with LY294002, with marginal significance (Figure 3(d)). The concentration of SDF-1*α* in cultured medium was significantly higher in early EPCs treated with the highest dose of cilostazol (30 *μ*M) as compared to other doses (Figure 3(e)).

These results may provide a mechanism by which cilostazol enhances proangiogenesis in human early EPCs, with SDF-1*α*/CXCR4 playing a significant role.

### 3.5. Hybrid Therapy with Administration of Cilostazol and Transplantation of Human Early EPCs Improved Blood Flow Recovery and Increases Capillary Densities in Murine Hindlimb Ischemia

**Figure 4:**
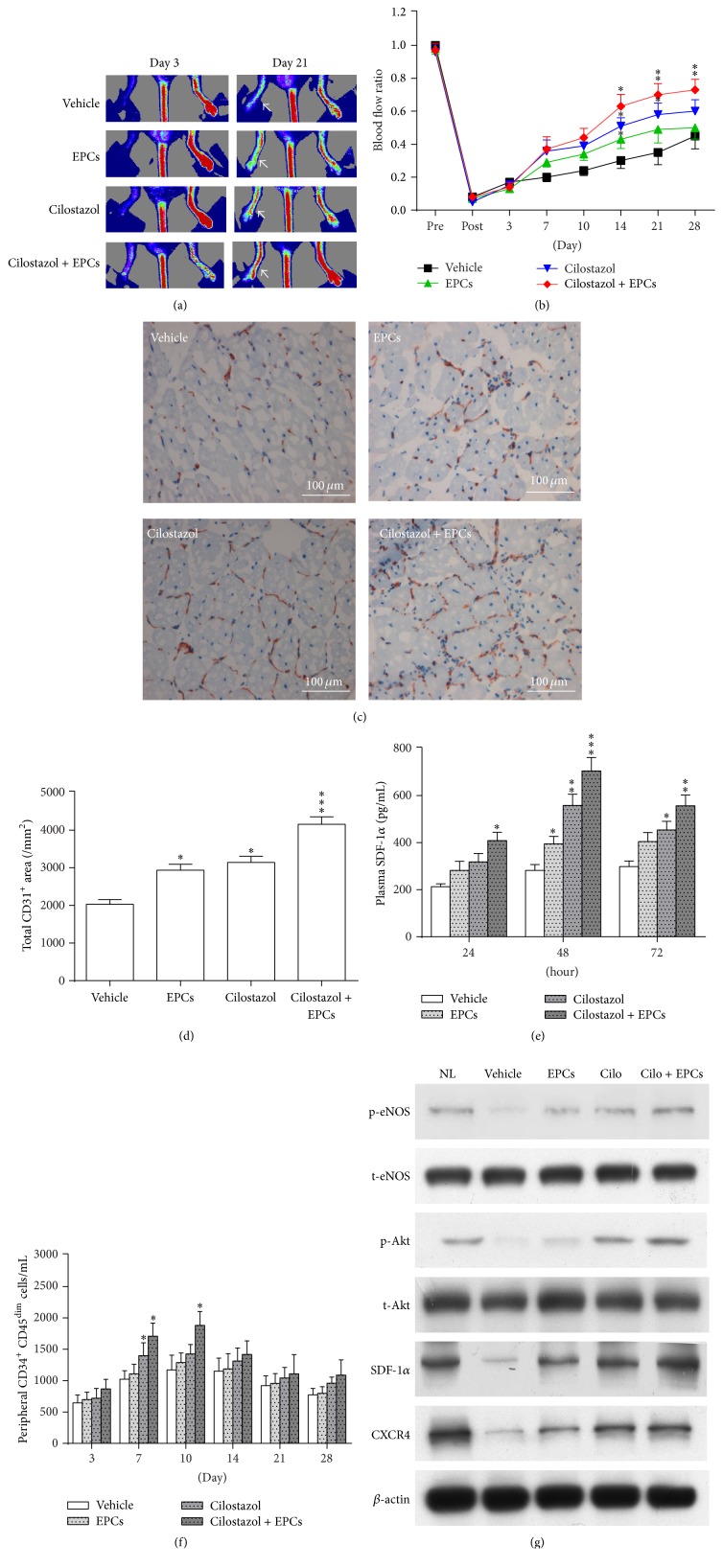
Different treatment strategies in mice with hindlimb ischemia. (a) Representative images of laser Doppler perfusion flow in hindlimbs. Cilostazol, EPC transplantation, and hybrid therapy accelerated flow recovery 14 days after surgery, in particular, with hybrid therapy (a, b). (c) Representative photos of capillaries in the leg muscles visualized by anti-CD31 immunostaining (red) and nuclei counterstained with hematoxylin (blue). Capillary density was significantly higher in EPCs-, cilostazol-, and, notably, combined EPCs and cilostazol-treated mice (d). (e) Measurement of plasma levels of SDF-1*α*. Plasma SDF-1*α* levels were significantly higher in all 3 active treatment groups as compared to the vehicle group 48 hours after surgery. However, the greatest effect was achieved and maintained during the 72-hour course in the hybrid group. (f) Quantification of peripheral CD34^+^CD45^dim^ cells in mice treated with different therapeutic strategies at different time points. (g) Phosphorylation of Akt/eNOS signaling molecules and expression of SDF-1*α*/CXCR4 proteins. Active treatments, especially hybrid therapy, could upregulate SDF-1*α*/CXCR4/Akt/eNOS molecules in ischemic hindlimbs. ^*∗*^ *P* < 0.05, ^*∗∗*^ *P* < 0.01, and ^*∗∗∗*^ *P* < 0.001, significantly different compared with vehicle-treated mice. CXCR4, C-X-C chemokine receptor type 4; eNOS, endothelial nitric oxide synthase; EPCs, endothelial progenitor cells; NL, nonligated; SDF-1*α*, stromal cell-derived factor-1*α*.

Hindlimb blood flow was monitored for up to 28 days after surgery by LDPI. Although administration of cilostazol or transplantation of human early EPCs alone could accelerate blood flow recovery at 14 days after surgery as compared to vehicle, the significant effects gradually vanished thereafter (Figures 4(a) and 4(b)). However, combined treatment of cilostazol and human early EPCs had the greatest improvement in limb perfusion, maintaining effects until 28 days after the operation compared to vehicle (Figures 4(a) and 4(b)).

Immunohistochemical staining of mouse CD31 in muscle sections revealed the presence of capillary ECs and was used as a marker of capillary density [4, 12]. Capillary density in the ischemic muscles 28 days after induction of hindlimb ischemia was significantly higher in the mice treated with hybrid therapy, cilostazol alone, and EPCs alone compared to vehicle (Figures 4(c) and 4(d)). Hybrid therapy had the most significant effect. Immunofluorescence staining showed that cotreatment with cilostazol and EPCs enhanced EPC engraftment and incorporation into neovascularization sites (Supplement 1 in Supplementary Material available online at http://dx.doi.org/10.1155/2016/3639868).

### 3.6. Cilostazol Increased Plasma Levels of SDF-1*α* and Circulating CD34^+^CD45^dim^ Cells

Our data revealed that only hybrid therapy with cilostazol and EPCs could significantly increase plasma SDF-1*α* level 24 hours after surgery (Figure 4(e)). Plasma SDF-1*α* levels were significantly higher in all 3 active treatment groups as compared to the vehicle group 48 hours after operation, whereas the greatest effect was achieved and maintained through 24 to 72 hours in the hybrid group (Figure 4(e)).

Increased CD34^+^CD45^dim^ cells in peripheral circulation might represent enhanced mobilization and viability of bone marrow-derived stem cells and be important in vasculoangiogenesis after ischemia and tissue injury [19, 20]. Herein, we measured and compared the number of circulating CD34^+^CD45^dim^ cells among different treatment groups in a murine hindlimb ischemia model by using flow cytometry. Both the cilostazol- and combined cilostazol and early EPCs-treated mice exhibited a time-dependent increase in the number of CD34^+^CD45^dim^ cells in the peripheral blood after induction of hindlimb ischemia, peaked on day 7 in the cilostazol group and reached a peak on day 10 after surgery in the hybrid group (Figure 4(f)). However, only marginally significant effect was observed in the EPC-treated mice (Figure 4(f)). Taken together, these results imply that hybrid therapy might have a beneficial effect on vasculoangiogenesis* in vivo*. Circulating CD34^+^CD45^dim^ cells could play a role.

### 3.7. The Effects of Different Treatment Strategies on Phosphorylation of Relevant Signaling Molecules and Expression of SDF-1*α* and CXCR4 Proteins in Ischemic Limbs

The phosphorylation of Akt/eNOS signaling molecules and the expression of SDF-1*α*/CXCR4 proteins were attenuated in ischemic muscle as compared to nonischemic regions (Figure 4(g)). Active treatments, especially hybrid therapy, could upregulate SDF-1*α*/CXCR4/Akt/eNOS molecules in ischemic hindlimbs (Figure 4(g)).

### 3.8. Cilostazol Could Provide a Synergistic Angiogenesis Effect with EPCs

**Figure 5:**
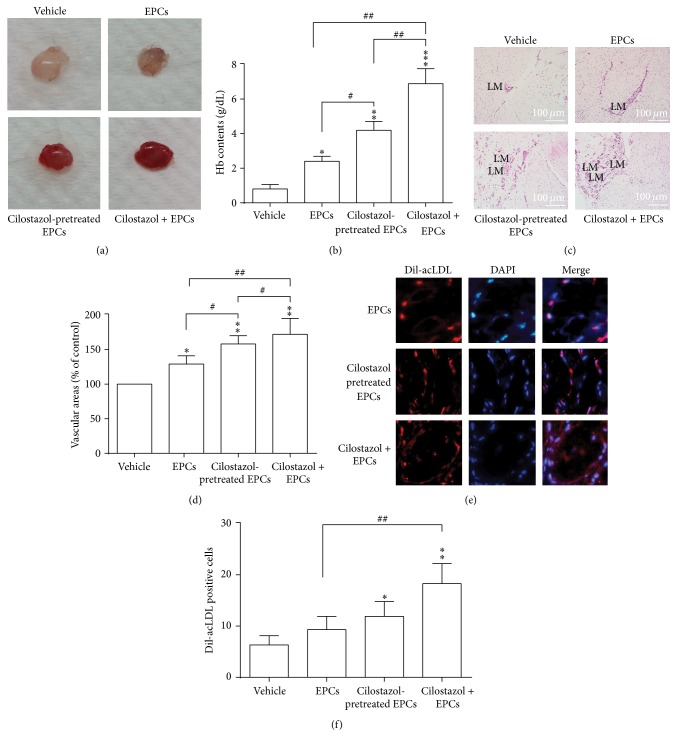
Murine Matrigel angiogenesis model. (a) Representative photos of Matrigel plaques. All Matrigel plaques with active treatment components inside have higher hemoglobin content as compared to plaques containing only the vehicle control (a, b). Matrigel plaques with cilostazol-pretreated EPCs inside contain more hemoglobin than those with EPCs alone (a, b). The highest hemoglobin content is seen in Matrigel plaques with cilostazol and EPCs (a, b). (c) Histological examination of neovascularization in Matrigel plaques by hematoxylin and eosin staining. All Matrigel plaques with active treatment components inside have larger areas of neovascularization than plaques containing only the vehicle control (c, d). Matrigel plaques with cilostazol-pretreated EPCs inside have significantly higher number of vascular areas than those with EPCs alone (c, d). (e) Immunofluorescence staining of implanted EPCs. A higher number of DiI-acLDL-prelabeled cells (red) counterstained with DAPI (blue) are found in Matrigel plaques containing cilostazol-pretreated EPCs and combination of cilostazol and EPCs (e, f). ^*∗*^ *P* < 0.05, ^*∗∗*^ *P* < 0.01, and ^*∗∗∗*^ *P* < 0.001, significantly different compared with vehicle-treated mice. ^#^ *P* < 0.05 and ^##^ *P* < 0.01, significantly different between active treatment groups. EPCs, endothelial progenitor cells; Hb, hemoglobin; DAPI, 4′,6-diamidino-2-phenylindole; DiI-acLDL, DiI-acetylated low density lipoprotein; LM, lumen.

In the murine Matrigel angiogenesis model, our data showed that transplantation of early EPCs pretreated with cilostazol* ex vivo* had a significantly higher hemoglobin content (Figures 5(a) and 5(b)) and more vascular formation (Figures 5(c) and 5(d)) in the Matrigel plaque than untreated EPC transplantations. Combined treatment had the greatest stimulating effects (Figures 5(a)–5(d)). Immunofluorescence staining showed that the number of DiI-acLDL positive cells, indicative of engrafted transplanted EPCs, was significantly higher in cilostazol-pretreated EPCs alone and in combined treatment of cilostazol and EPCs (Figures 5(e) and 5(f)). Transplantation of cilostazol-pretreated EPCs and hybrid therapy showed more incorporation of EPCs into neovascularization sites than EPCs alone (Figure 5(e)). To sum up, these findings imply that cilostazol could provide a synergistic proangiogenic effect of EPCs.

### 3.9. The Stimulatory Effect of Cilostazol on Proangiogenesis Functions in EPCs Could Be Modulated by SDF-1*α In Vitro*

**Figure 6:**
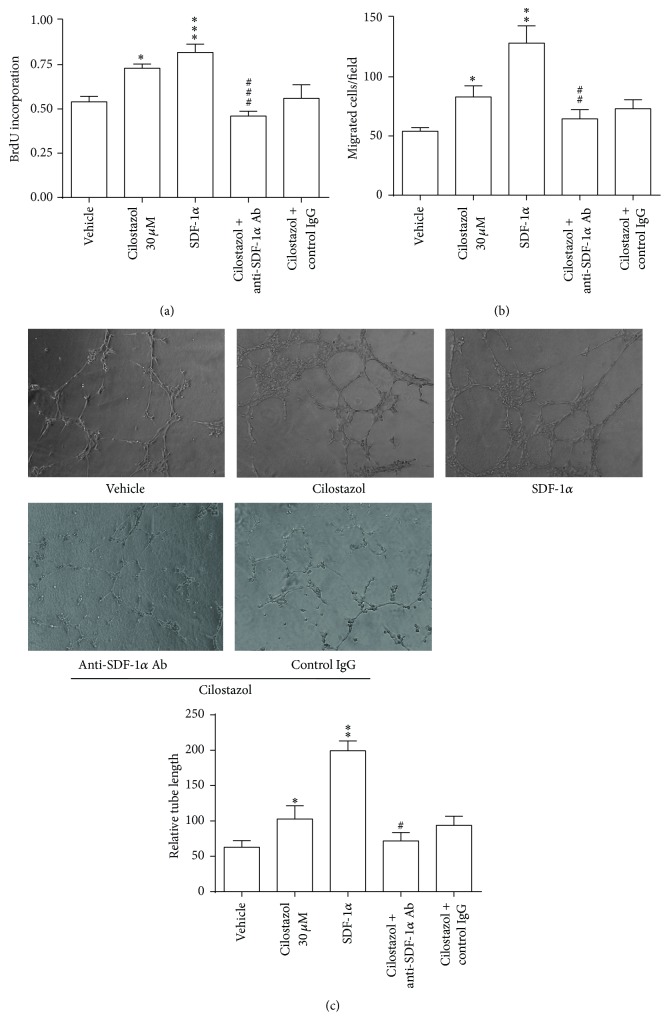
The role of SDF-1*α* in the stimulatory effect of cilostazol on proangiogenesis functions in human early EPCs. Anti-SDF-1*α* neutralizing antibody could significantly suppress the effect of cilostazol on proliferation (a), migration (b), and* in vitro* vascular tube formation of EPCs (c). ^*∗*^ *P* < 0.05, ^*∗∗*^ *P* < 0.01, and ^*∗∗∗*^ *P* < 0.001, significantly different compared with vehicle-treated cells. ^#^ *P* < 0.05, ^##^ *P* < 0.01, and ^###^ *P* < 0.001 versus cilostazol-treated (30 *μ*M) cells. Ab, antibody; BrDU, 5-bromo-2′-deoxyuridine; IgG, immunoglobulin type G; SDF-1*α*, stromal cell-derived factor-1*α*.

Human early EPCs were incubated with cilostazol (30 *μ*M) plus anti-SDF-1*α* neutralizing antibody (10 mg/mL) for 24 hours in the proliferation and migration experiments, and for 3 days in vascular tube formation experiments. Anti-SDF-1*α* neutralizing antibody significantly attenuated the effect of cilostazol on proliferation, migration, and* in vitro* vascular tube formation of EPCs (Figures 6(a)–6(c)).

### 3.10. The Additive Effect of Cilostazol on EPC Transplantation Could Be Modulated by SDF-1*α In Vivo*

**Figure 7:**
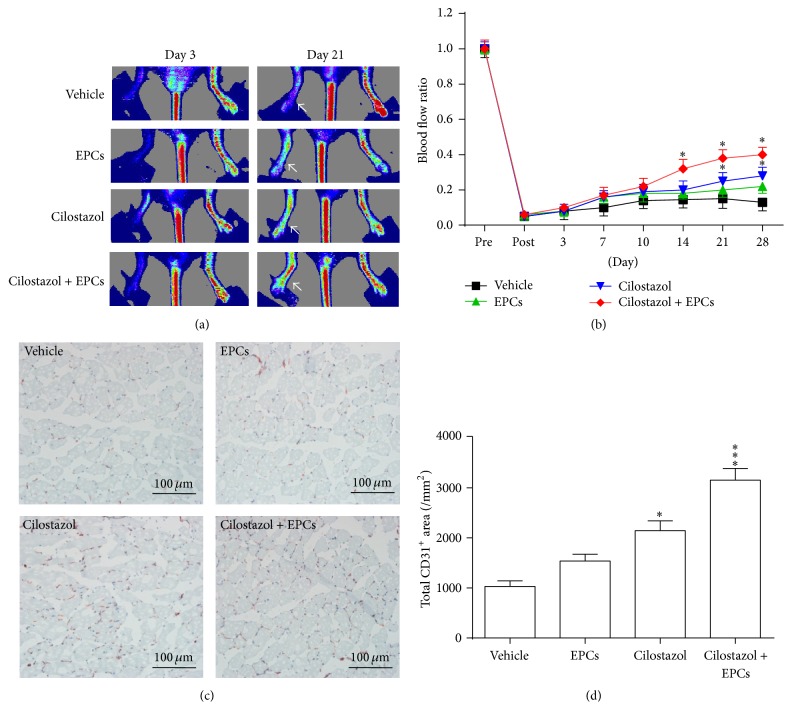
*In vivo* inhibition of SDF-1*α* in mice with hindlimb ischemia. (a) Representative images of laser Doppler perfusion flow in hindlimbs of mice (indicated by arrows). Cilostazol and hybrid therapy with cilostazol and EPCs both accelerated flow recovery 21 days and 28 days after surgery, whereas EPC transplantation alone did not have a significant effect (a, b). (c) Representative photos of capillaries in the leg muscles visualized by anti-CD31 immunostaining (red) and nuclei counterstained with hematoxylin (blue). Capillary density was significantly higher in cilostazol-, and, notably, combined EPCs and cilostazol-treated mice (d). ^*∗*^ *P* < 0.05 and ^*∗∗∗*^ *P* < 0.001, significantly different compared with vehicle-treated mice. EPCs, endothelial progenitor cells.

In the murine hindlimb ischemia model, the beneficial effects of 3 active treatments on flow recovery and neovascularization in the ischemic limb were all significantly attenuated with IM injection of an anti-SDF-1*α* neutralizing antibody (Figures 7(a)–7(d)).

## 4. Discussion

**Figure 8:**
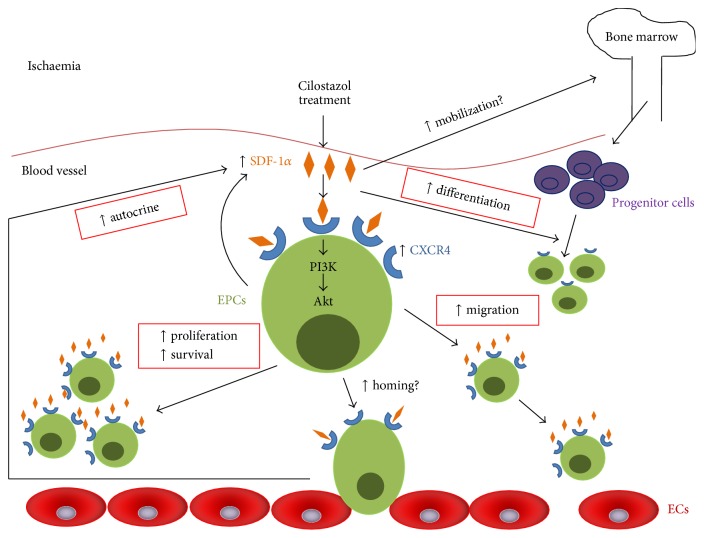
Schematic representation of the effects of cilostazol on proangiogenic functions of human early EPCs mediated by activation of the SDF-1*α*/CXCR4 system. EPCs are in green, bone marrow-derived progenitor cells are in purple, and vascular ECs are in red. CXCR4, C-X-C chemokine receptor type 4; ECs, endothelial cells; EPCs, endothelial progenitor cells; PI3K, phosphoinositide 3-kinase; SDF-1*α*, stromal cell-derived factor-1*α*.

Our data show that cilostazol promotes a large number of proangiogenic functions in human early EPCs, and hybrid therapy with cilostazol and human early EPCs provide an additive effect in murine hindlimb ischemia and a synergistic effect in murine Matrigel angiogenesis. Cilostazol treatment enhanced differentiation of human early EPCs toward the endothelial lineage. In addition, cilostazol-induced colony formation, proliferation, and migration, inhibited apoptosis, increased formation of vascular tubes* in vitro*, upregulated the expression of SDF-1*α* and CXCR4 proteins, and promoted secretion of SDF-1*α* from human early EPCs. Furthermore, coadministration of cilostazol and transplantation of human early EPCs promoted vasculoangiogenic activity during hindlimb ischemia. Hybrid therapy stimulated the most effective release of SDF-1*α* and increased circulating bone marrow-derived stem cells in mice with hindlimb ischemia. Cilostazol treatment could provide a synergistic angiogenesis effect with EPCs in murine Matrigel angiogenesis model. Finally, the stimulatory effect of cilostazol on the proangiogenic functions in human early EPCs, and the additive effect of cilostazol and human early EPCs in murine hindlimb ischemia, could be mediated through activation of SDF-1*α*/CXCR4/PI3K/Akt signaling pathway (Figure 8). Our data may have substantial clinical implications.

The effect of cilostazol on the proangiogenesis functions of human early EPCs is not well understood. Our previous studies [4, 12] showed that cilostazol may increase colony formation by human early EPCs. The current study extensively evaluated the proangiogenesis functions of human early EPCs modulated by cilostazol. Cilostazol increased colony formation, proliferation, and migration, inhibited apoptosis, and increased formation of vascular tubes* in vitro,* as well as enhanced EPC engraftment and incorporation to neovascularization sites. This suggests that cilostazol could provide a vasculoangiogenesis effect as well as helping EPCs to overcome harsh ischemic environments. This speculation is supported by our previous [4, 12] and current* in vivo* experiments. Our previous studies performed in ICR mice with [12] or without [4] hyperglycemia revealed that cilostazol could significantly increase circulating number of CD34^+^CD45^dim^ cells in peripheral blood. Our current study showed coadministration of cilostazol and IM transplantation of human early EPCs in SCID mice had the optimal effect on the circulating number of CD34^+^CD45^dim^ cells and plasma levels of SDF-1*α*. This indicated an improved vasculoangiogenesis effect of cilostazol on bone marrow-derived stem cells. EPCs are known to have both paracrine and autocrine effects [21, 22]. Our data further confirmed that cilostazol-induced beneficial effects* in vitro* and* in vivo* could be mediated through activation of the SDF-1/CXCR4 system and promoted secretion of SDF-1*α* from transplanted human EPCs. The SDF-1/CXCR4 system has been reportedly involved in proliferation, migration, and antiapoptosis of CD34^+^ cells* in vitro* [23, 24]. Lataillade et al. have shown that cilostazol could enhance the function of intrinsic bone marrow-derived EPCs in rat for reendothelialization in a rat carotid balloon injury model partly mediated by activation of the SDF-1/CXCR4 system [24]. They did not examine the additive or synergistic effects of cilostazol on transplanted human EPCs in murine hindlimb ischemia, Matrigel angiogenesis models, nor the role of the SDF-1/CXCR4 system on the underlying mechanism. Our current study demonstrated that cilostazol promoted proangiogenic functions in human early EPCs* in vitro* and* in vivo* partly mediated through activation of the SDF-1/CXCR4 system. Accordingly, SDF-1*α* is positively regulated by stem cells or progenitor cells, and* vice versa*, to form a positive feedback loop. Cilostazol enhanced the SDF-1*α*-progenitor loop. The current findings were consistent with our previous randomized-controlled trial [15] showing that cilostazol could increase circulating EPCs and plasma levels of SDF-1*α*, and changes of both after treatment were correlated significantly in patients with peripheral artery disease.

Previous studies in many cell types have implicated the PI3K/Akt signal transduction pathway as the control in directing cell migration and sensing of chemoattractant gradients by cells [25]. PI3K/Akt signaling may work as one of the downstream pathways of the SDF-1/CXCR4 system [25, 26]. Our current study verified the promoting effect of cilostazol on proangiogenesis in human early EPCs through the SDF-1/CXCR4/PI3K/Akt signaling pathway.

Several strategies have been developed to enhance cell therapy efficiency in hindlimb ischemia [2] such as coadministration of vasculoangiogenic factors [2, 27], modulation of the relevant signaling pathways to facilitate efficient differentiation [2, 28], genetic engineering using biodegradable nanoparticles before transplantation [2, 29], and modification of delivery strategy by scaffolds seeded with stem cells [2, 29]. Although many progenitor/stem cells obtained from patients have been investigated and are alleged to be suitable for autologous transplantation, their therapeutic application is still limited [2]. Our current study demonstrated that incubation of early EPCs with cilostazol* ex vivo* could improve vasculoangiogenesis as compared to transplantation of only EPCs in a murine Matrigel angiogenesis model, suggesting a synergistic effect of cilostazol. Our study has revealed the potential therapeutic role of combined cilostazol administration and EPC transplantation in patients with peripheral artery disease, especially in CLI. However, cilostazol is only indicated for the reduction of symptoms of intermittent claudication, with successful treatment indicated by an increase in walking distance. According to our previous [15] and current studies, this compound might be considered for use in patients who have peripheral artery disease, especially, with CLI undergoing cell therapy.

Being even more complex and heterogeneous in nature, circulating EPCs are traditionally classified into 3 major cell types [22, 30, 31] which have been developed according to their morphology and time-dependency in culture. Despite this, both early and late EPCs showed comparable* in vivo* vasculogenic capacity; early EPCs secreted angiogenic cytokines more so compared to late EPCs during* in vitro* culture [30]. Moreover, CFU-Hill cells showed low neovascularization potential* in vivo* [31]. Therefore, we chose human early EPCs for transplantation and proved its efficacy in vascular regeneration.

In conclusion, cilostazol promotes a large number of proangiogenic functions in human early EPCs through activation of the SDF-1/CXCR4/PI3K/Akt signaling pathway. Hybrid therapy with cilostazol and human early EPCs provided an additive effect in murine hindlimb ischemia and a synergistic effect in murine Matrigel angiogenesis models. Cilostazol enhances the SDF-1*α*-progenitor loop. Our results suggest that the hybrid therapy with cilostazol and EPCs may provide beneficial effects in patients with ischemic disease.

## Supplementary Material

Human EPCs incorporated into neovascularization sites in ischemic hindlimb muscles.
