# Supplementary material for: Cilostazol Improves Proangiogenesis Functions in Human Early Endothelial Progenitor Cells through the Stromal Cell-Derived Factor System and Hybrid Therapy Provides a Synergistic Effect In Vivo
Source: Biomed Res Int. 2016 Aug 9;2016:3639868. doi: 10.1155/2016/3639868 (PMC4993925; doi:10.1155/2016/3639868)
Supplement: Supplementary file 1 — Human EPCs incorporated into neovascularization sites in ischemic hindlimb muscles. [file 3639868.f1.doc]

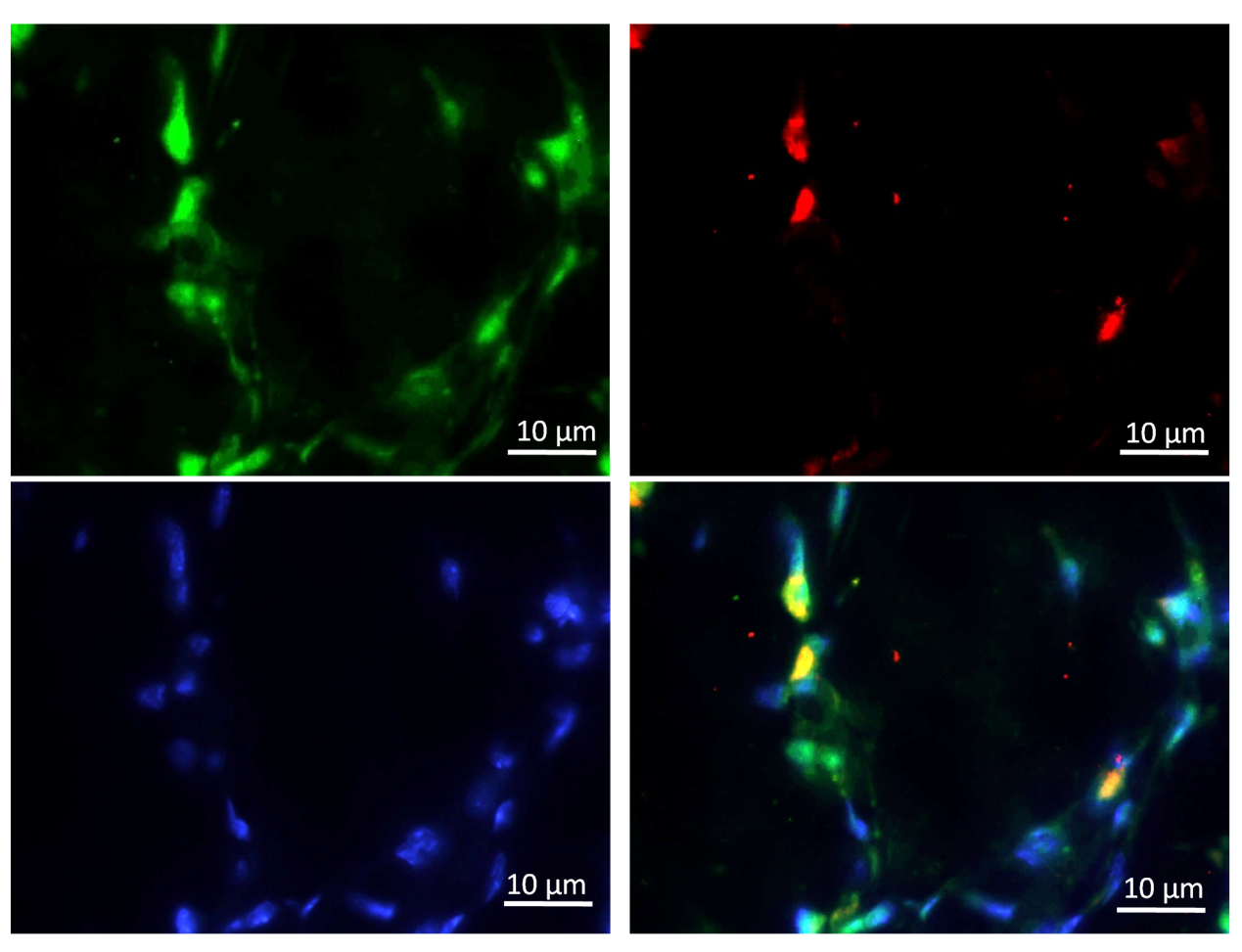


**Supplemental FIGURE 1:** Human EPCs incorporated into neovascularization sites in ischemic hindlimb muscles. Immunofluorescence staining shows mouse vascular endothelial cells (green) (left upper panel) counterstained with DAPI (blue) (left lower panel) in vasculatures. DiI-acLDL-labeled human early EPCs are present in a vascular cluster (red) (right upper panel). More human EPCs are co-localized with mouse vascular endothelial cells in mouse with hybrid therapy (merge) (right lower panel). DAPI, 4',6-diamidino-2-phenylindole; DiI-acLDL, DiI-acetylated low density lipoprotein; EPCs, endothelial progenitor cells.
